# The challenge of achieving vitamin D adequacy for residents living in long-term care

**DOI:** 10.1017/S136898002100238X

**Published:** 2022-01

**Authors:** Allison L Cammer, Susan J Whiting

**Affiliations:** College of Pharmacy and Nutrition, University of Saskatchewan, 107 Wiggins Road, Saskatoon, SK S7N 5E5, Canada

In this issue, Robbins and co-workers report a study of vitamin D supplement use in long-term care (LTC) homes in the USA^(1)^ that is an important reminder of the need to consider how those living in sun-deprived situations can meet their need for vitamin D, especially those with higher dietary recommendations than the rest of the population due to their age and/or medical conditions. These investigators used a natural experiment to determine the type and content of supplements that successfully met the vitamin D needs of LTC residents. However, as will be described, allowing residents in LTC to choose the type of vitamin D supplements can have a hit-or-miss outcome. Robbins *et al.* show the evidence for this with only 43 % of residents consuming 20 μg/d which was calculated as necessary to maintain adequacy, while 9% of residents risked consuming greater than the Upper Level of 100 μg/d which is more than the intake needed to maintain adequacy.

LTC facilities are essential components of elder care in Western countries. LTC homes (also called nursing homes, skilled nursing facilities and care homes) are residential care facilities for those who can no longer independently manage activities of daily living and include health and social care provision. A spectrum of LTC homes exists, including private and public funded as well as for-profit and not-for-profit status^(2)^. Care is predominantly supported by care aides (also called continuing care assistants, nursing assistants and personal support workers) who are ‘unregulated’, i.e. do not have professional status or certified credentials. Residents have access to medical care via physician, nurse and allied provider services (e.g. dietitian, physical and occupational therapist and speech-language pathologist) who are regulated or trained certified providers. A standard of care is typically legislated federally or by state/province, for example, in the USA by the Centers for Medicare & Medicaid Services^(3)^. Assessment of nutritional status and needs as well as provision of an appropriate and acceptable diet that satisfies dietary requirements is often required, inclusive of those residents requiring a therapeutic diet or texture modification. Nevertheless, the LTC facilities’ financial status can lead to limitations that reduce or prevent residents from achieving optimal dietary intake and nutrition care. Combined with the increased level of acuity of resident healthcare needs and comorbid conditions, malnutrition is a common issue among elderly residents in LTC homes^(4)^.

Widespread public health awareness of vitamin D deficiency in the USA was not recognised as a particularly serious problem for older adults until the 1997 Dietary Reference Intake report from the Institute of Medicine^(5)^. The 1997 recommendations for adults 50 to 70 years of age were doubled from a previous 1985 Recommended Dietary Allowance (RDA) of only 5 μg/d^(6)^ to 10 μg/d and tripled to 15 μg/d for adults over 70 years. The reason for the change in 1997 was due to compelling evidence that the vitamin D status of older adults was much lower than expected. Researchers found very low circulating levels of 25-hydroxyvitamin D (25(OH)D), the main indicator of vitamin D status^(5)^, in homebound and institutionalised older adults even when they consumed vitamin D at levels of 10 μg/d, a level which was twice the 1985 RDA^(7)^.

In 2011, the National Academy of Sciences Engineering and Medicine (NAM, formerly, IOM) set RDAs at an even higher levels, with all ages between 1 and 70 years having an RDA of 15 μg/d and those over 70 years having an RDA of 20 μg/d^(8)^. It has been recognised that older adults have increased needs due to metabolic inefficiencies in vitamin D metabolism and skin synthetic capacity^(8)^. In agreement with this notion, many other government bodies around the world have also made recommendations for vitamin D since 2011, and most range between 15 and 20 μg/d for adults > 75 years of age^(9)^. The target 25(OH)D level for the recommendations also vary, most following what the NAM 2011 report determined to represent sufficiency which is 25(OH)D concentrations of 50 nmol/l^(8)^. However, a higher 25(OH)D level set at 75 nmol/l is used by the Endocrine Society^(10)^ based on evidence of greater need in older adults who are at greater risk for chronic diseases; other organisations have made similar recommendations^(11)^ and put forth the argument that in situations where the person is not healthy (using medications compromising vitamin D metabolism or suppressing immune response, or having serious comorbidities), a higher intake is justified. Table 1 shows the recommended levels of vitamin D intake for older men and women in various countries and those recommended by different professional societies impacting the health of the older adults.

**Table 1:** Selected interpretive criteria for measurement of 25-hydroxyvitamin D (25(OH)D) and corresponding Recommended Dietary Allowance (RDA) (or equivalent) for the oldest adult group. Public health is to maintain health in already healthy; clinical health is for those at-risk*

Organisation/Country (date of issue)	Cut-off 25(OH)D	RDA or equivalent for > 75 years
Public health	nmol/l	μg/d
NAM (2011)	50	20†
Nordic (2012)	50	20
DACH (2012)	50	20‡
Netherlands (2012)	30	20‡
Australia–NZ (2013)	50	20
United Kingdom (2016)	25	10
EFSA (2016)	50	15
Clinical health		
Int Osteoporosis Fnd (2010)	75	50‡
Endocrine Soc (2011)	75	37·5–50
American Geriatric Soc (2014)	75	100‡

RDA = recommended dietary allowance; NAM = National Academy of Sciences Engineering and Medicine, formerly Institute of Medicine; Soc = Society; Int = International; Fnd = Foundation; DACH = German-speaking countries of Germany, Austria and Switzerland; EFSA = European food safety authority.

* Adapted from Bouillon^(9)^.

† This intake also applies to > 70 years.

‡ This intake also applies to > 65 years.

In the intervening 30 years since the report by Gloth and colleagues^(7)^ questioned how older adults could maintain vitamin D levels while housebound, much has been learned about vitamin D’s roles in both skeletal and non-skeletal health^(10,12)^. Adequacy of dietary vitamin D intake needs to consider that it is not just bone health that is impacted by vitamin D deficiency. Despite this consensus in many government recommendations for an RDA at or approaching 20 μg/d, meeting the vitamin D needs of older adults living in LTC remains a concern. To provide adequate vitamin D status, three strategies can be employed: sun (or more specifically ultraviolet B radiation) exposure for skin synthesis; dietary intake of natural and/or fortified foods and oral supplements. Here, we consider the efficacy of each of these strategies in the context of ensuring adequate vitamin D for older adult residents of LTC.

Skin synthesis of vitamin D is a natural way to meet vitamin D needs, yet there are concerns for this as a consistent and effective method for residents of LTC who by definition are ‘homebound’ and who are mostly older. Irradiation with sun or ultraviolet B radiation has been studied in LTC homes with limited success. Sun exposure can raise vitamin D levels in older adults, but it is well established that efficiency falls by 13 % per decade^(13)^. Residents with dark skin pigmentation would require impractical longer exposure times for meaningful vitamin D synthesis^(12)^. For those homes in areas where winter lasts many months, there would be long periods of time without adequate sun exposure. For those homes in particularly temperate areas, heat exposure would be a concern, particularly during the time of day when there is adequate ultraviolet B radiation for skin synthesis of vitamin D. A recent pilot study in the USA^(14)^ found no significant improvement in vitamin D status upon implementing sun exposure to residents of LTC. Another study testing timed ultraviolet B radiation-light exposure did see improvements in circulating 25(OH)D but concluded ‘in our experience [it was] time-consuming for the ward staff and thus less convenient than oral vitamin D supplementation’^(15)^. Thus, to meet vitamin D needs of LTC residents, intake from foods and/or supplements is preferred.

Many researchers report that dietary intakes from natural or fortified foods cannot meet dietary recommendations, except in a few unusual circumstances where ocean fish and sea mammals are consumed following cultural traditions such as those practiced in Greenland^(16)^. It is possible to achieve 25(OH)D concentrations defining adequacy (cut-off values of > 75 nmol/l for older adults) from natural foods; however, as shown by Robbins and colleagues in this issue^(1)^, even in a country such as the USA where foods are fortified with vitamin D, intake of vitamin D through food alone does not ensure meeting the RDA of 20 μg/d for adults over 70 years. In their analysis of menus provided in five LTC residences in Texas, Robbins *et al.*^(1)^ found that the average amount of vitamin D offered (but not necessarily ingested) at meals was 8 μg/d. In the LTC homes they studied, the authors report 38 % of the residents did not take vitamin D supplements, and this amount of vitamin D in their food was not adequate to maintain 25(OH)D at 75 nmol/l for four-fifths of the residents in this nonuser group. Thus, food alone is not an effective strategy for residents of LTC, even in countries allowing vitamin D fortification of a variety of food categories.

On first consideration, dietary supplement use appears to be an effective strategy to improve vitamin D status in older LTC residents; but questions about the practical application of this strategy remain. It is noteworthy that Robbins and co-workers found that only two-thirds of the LTC residents took vitamin D supplements and most who took only a multivitamin or a Ca supplement containing vitamin D (Ca+D) consumed an insufficient dose of vitamin D. The most effective supplement was vitamin D as the only ingredient. In the USA, vitamin D supplements are sold over-the-counter as tablets, caplets or drops containing varying levels of vitamin D between 10 μg and 250 μg^(17)^ explaining why a stand-alone vitamin D supplement could be effective in raising 25(OH)D levels above the target 25(OH)D concentration. The second effective method of supplement choice was taking a combination of supplements, at least two of: multi-vitamin, Ca+D or D-alone. This allowed supplements to be additive. Of residents, only 43 % had intakes > 20 μg which in subsequent analysis showed was adequate for most to achieve the desired 25(OH)D concentration cut-off. This is significant as the recommended intake for older adults by most organisations is 20 μg/d (Table 1).

A concern not emphasised by Robbins *et al.*^(1)^ was that a small group of residents over-consumed vitamin D using supplements. There were 9 % of residents whose intake from supplements exceeded the Upper Level for vitamin D, which is 100 μg in the USA^(8)^ and Europe^(18)^. Importantly, further analysis by Robbins et al showed that taking a lower supplement dose, between 50 and100 μg, was just as effective as taking a dose > 100 μg. Therefore, if residents knew higher doses were not needed, this could reduce the risk for adverse effects.

While effective, there are concerns about ad hoc supplement use for maintaining vitamin D status of residents in LTC. First, not all residents know to take supplemental vitamin D and encouragement for supplement use may be lacking. Second, many residents who take supplemental vitamin D do not take enough. Residents in the study in this issue^(1)^ were taking multivitamins or Ca supplements and may not be knowingly taking vitamin D. And third, there is the risk of exceeding the Upper Level, which can increase the risk of adverse effects as well as prove to be an unnecessary cost.

An important gap in our ability to implement an effective approach needed to maintain vitamin D status among older adult residents is the lack of an integrated system of vitamin D supplement use in place across LTC facilities. The American Geriatrics Society^(19)^ provides an initial step towards this goal which is an algorithm designed to address the needs of individual residents to ensure adequate vitamin D intake. More importantly, creative thinking is needed to develop the next steps in creating a viable integrated system for vitamin D supplementation that would be unique to the needs of older LTC residents. A possible next step could be to apply this institutionally, using supplemental sources of vitamin D such as drops added to residents’ meals or snacks rather than taken in a pill-like form akin to a medication, which for LTC residents can lead to concerns of polypharmacy^(20)^. Dietitians are health professionals trained to assess dietary intakes from both food and supplement sources and would be capable of enhancing foods with vitamin D without altering menu choices, as the latter option adds costs and may incur culturally inappropriate foods being offered. With the evidence mounting that vitamin D plays an important role in immune protection against communicable diseases and risk lowering in a number of chronic diseases, it is fitting that older, vulnerable adults receive vitamin D in amounts that make a beneficial difference to their health outcomes^(21)^.
